# Uncommon Case of Uterine Rupture Associated With Splenic Artery Aneurysm and Rupture During Pregnancy: A Clinical Report and Review of the Literature

**DOI:** 10.1002/ccr3.71743

**Published:** 2026-03-01

**Authors:** Behnaz Pazoki, Mahtab Motevasselian, Mohsen Khaleghian, Roya Biglarifard, Seyed Hamzeh Mousavie

**Affiliations:** ^1^ Firoozgar Clinical Research Development Center (FCRDC) Department of Obstetrics and Gynecology, School of Medicine, Firoozgar Hospital Tehran Iran; ^2^ Department of General Surgery, School of Medicine Hazrat ‐Rasool General Hospital Iran University of Medical Sciences Tehran Iran

**Keywords:** intra‐abdominal hemorrhage, pregnancy complication, splenic artery aneurysm, uterine rupture

## Abstract

We present a case of concomitant splenic artery aneurysmal rupture with uterine rupture in a 32 year‐old pregnant woman at 34 weeks of gestation with a history of one previous Cesarean section. The patient presented to the Emergency Department (ED) due to a sudden abdominal pain, which resolved spontaneously. In the ED, she displayed tachycardia, sweating, and lethargy, with no detectable fetal heart rate, raising suspicion of uterine rupture. Surgical exploration revealed significant intra‐abdominal hemorrhage from a prior uterine incision, resulting in intrauterine fetal demise (IUFD) and placental abruption. The uterine rupture was promptly repaired, and the uterus remained stable. Subsequently, substantial bleeding and a massive hematoma were discovered. The patient received blood transfusions and underwent surgery to address the source of bleeding, identified as a ruptured splenic artery aneurysm, and successfully managed with packing and ligation. She recovered in the Intensive Care Unit (ICU). This case highlights the rare co‐occurrence of uterine rupture and splenic artery aneurysm without apparent changes in vital signs or abdominal findings. In spite of the IUFD and massive hemorrhage, the patient ultimately maintained normal blood pressure.

## Key Clinical Message

Simultaneous uterine rupture and splenic artery aneurysm rupture during pregnancy is a rare but life‐threatening emergency. Early recognition and prompt surgical intervention are crucial to prevent severe maternal and fetal morbidity and mortality.

## 1 Introduction

Uterine rupture during pregnancy is a serious obstetric complication that can lead to significant maternal and fetal morbidity and mortality. The risk of uterine rupture increases during pregnancy, with recurrent exposure to high levels of oxytocin [1]. Splenic artery aneurysms, on the other hand, are uncommon vascular conditions that can be life‐threatening if they rupture [2]. Here, we describe a unique case involving the simultaneous occurrence of uterine rupture and splenic artery aneurysm rupture in a pregnant woman, with unusual clinical findings.

## 2 Case History/Examination

A 32 year‐old woman, gravida 2, para 1, live 1, with a history of one previous Cesarean section and no significant medical or medication history, presented to the Emergency Department (ED) after experiencing sudden abdominal pain that resolved spontaneously. At 34 weeks of gestation, she was referred to the ED due to tachycardia, sweating, and lethargy. Notably, her prenatal screening and sonography had been unremarkable throughout the pregnancy.

Upon arrival at the ED, fetal heart rate was not detectable, raising concerns of uterine rupture. Subsequent exploratory laparotomy revealed significant intra‐abdominal bleeding originating primarily from the site of her prior uterine incision. Tragically, the fetus was found to be intrauterine and deceased, accompanied by placental abruption. Prompt surgical repair of the uterine rupture was performed, leading to a well‐contracted, non‐bleeding uterus.

However, the patient continued to experience significant hemorrhage, with exploratory laparotomy revealing a massive hematoma extending from beneath the diaphragm to the duodenum. To address the substantial blood loss, the patient received 8 units of packed red blood cells and 4 units of fresh frozen plasma through a central venous catheter for hemodynamic support.

During the surgical intervention, the source of ongoing bleeding was traced to an aneurysm of the splenic artery. The ruptured splenic artery was meticulously packed and ligated bilaterally, effectively halting the hemorrhage. Following successful control of bleeding, the patient was transferred to the Intensive Care Unit (ICU) and ultimately made a full recovery.

## 3 Methods

In this case report, we examined a 32 year‐old pregnant woman at 34 weeks gestation who experienced sudden abdominal pain, followed by tachycardia, sweating, and lethargy. Despite unremarkable prenatal screenings, the absence of fetal heart rate upon presentation led to immediate exploratory laparotomy. Significant intra‐abdominal bleeding was identified, originating from a prior uterine incision, accompanied by placental abruption and an intrauterine fetal demise. The patient underwent prompt surgical repair of the uterine rupture, stabilizing the uterus.

Due to continued hemorrhage, further exploratory laparotomy revealed a massive hematoma and bleeding traced to a ruptured splenic artery aneurysm. Surgical intervention included meticulous packing and bilateral ligation of the ruptured splenic artery, followed by the administration of 8 units of packed red blood cells and 4 units of fresh frozen plasma for hemodynamic support. Postoperative care involved transfer to the Intensive Care Unit (ICU), where multidisciplinary management facilitated the patient's full recovery.

## 4 Discussion

This case presents a unique and rare clinical scenario in which uterine rupture and splenic artery aneurysm rupture occurred concomitantly during pregnancy. Despite the serious consequences of these events, the patient's vital signs remained within normal limits, her abdominal wall was firm, and the fetus was retained within the uterine cavity throughout the entire episode. However, the condition resulted in IUFD.

In our case, when the abdomen was opened, the uterus had ruptured from the previous incision and the fetus was intrauterine fetal demise (IUFD). After the repair of the uterine rupture, we still observed significant intra‐abdominal bleeding, not originating from the pelvis. Considering that the patient had no prior history of relevant problems and presented with severe headache and markedly elevated blood pressure just before admission, we suggest that the splenic artery aneurysm rupture may have been triggered by the acute blood pressure spike. The intraoperative findings and surgical timeline also confirm that the rupture was spontaneous and not iatrogenic.

Furthermore, the ruptured splenic artery aneurysm was diagnosed intraoperatively during exploratory laparotomy, after completion of the uterine repair. Given the emergent setting, the patient's hemodynamic instability, and the fact that the aneurysm was discovered during open surgery, immediate surgical repair was the only feasible approach. Endovascular treatment was not an option under these circumstances.

Spontaneous rupture of a splenic artery aneurysm is a rare occurrence during pregnancy, while it increases during the third trimester, similar to the timing observed in our case [3, 4, 5]. This condition typically leads to massive hemorrhage, resulting in significant consequences for both the mother and the fetus, with mortality rates ranging from 70% to 90% [2]. The precise etiology of such vascular events during pregnancy is unclear, with research implicating hormonal and physiological changes that affect blood volume, blood pressure, and cardiac output [3, 4, 5]. Other risk factors for splenic artery aneurysms include multiparity, portal hypertension, atherosclerosis, and fibrodysplasia [5]. According to the literature, 95% of splenic artery aneurysm cases during pregnancy are asymptomatic, which highlights the substantial nature of this condition [5].

Similar to our case, the clinical presentation of a ruptured splenic artery aneurysm is nonspecific and often characterized by acute abdominal pain, which is localized in the epigastrium or the left hypochondrium. Rapid and pronounced deterioration usually follows, accompanied by worsening vital signs and the onset of hemorrhagic shock. Differential diagnosis includes placental abruption, pulmonary embolism, uterine rupture, and the rupture of other arterial aneurysms [6]. This is important for physicians to consider this important and critical differential diagnosis in mind for similar cases.

This case underscores the critical association between splenic artery rupture and uterine rupture, which worsened the complexity of the diagnosis and management of this case. The survival rates of both the patient and the fetus in such cases depend on the timely detection and instant surgical intervention. Fortunately, our patient witnessed a successful outcome due to the prompt and precise collaboration between surgical and gynecological teams [7, 8].

In managing a ruptured splenic artery aneurysm (SAA), the unequivocal imperative is urgent surgical intervention following adequate fluid resuscitation. During surgery, the administration of tranexamic acid or adherence to massive transfusion protocols can be considered. When there is a suspicion of intraperitoneal bleeding, opting for a vertical incision is preferable over a Pfannenstiel incision. If an emergent delivery during surgery is necessitated, it is advisable to perform fetal removal following splenic artery ligation to alleviate the risk of potentially fatal secondary uterine bleeding [7, 8].

The risk of uterine rupture is usually increased during pregnancy, particularly in individuals with a history of uterine incision. Management of uterine dehiscence in a patient with a stable fetal heart rate tracing lacks a standardized approach. Full‐term pregnancies often warrant cesarean delivery in cases of uterine dehiscence, whereas expectant management has demonstrated efficacy when uterine dehiscence occurs in the preterm period [9].

It is essential to note that radiologic diagnostic tests often fall short in cases like these, and clinical suspicion remains a pivotal element in promptly identifying such conditions [10]. In our particular case, the patient's critical and unstable condition precluded the possibility of conducting additional imaging studies.

To the best of our knowledge, this is the first case of a splenic artery aneurysm rupture concomitant with uterine rupture. Intriguingly, despite the uterine rupture, the fetus safely remained within the uterus and did not cause further complications.

## 5 Conclusion and Results

Simultaneous uterine rupture and splenic artery aneurysm rupture during pregnancy, as described in this case, is exceedingly rare. Prompt recognition, surgical intervention, and multidisciplinary care were instrumental in achieving a successful outcome for the patient. This case underscores the importance of maintaining the suspicion and considering these uncommon etiologies in pregnant patients presenting with abdominal pain, as well as the challenges posed by managing such complex obstetric and vascular emergencies.

## Author Contributions

**Behnaz Pazoki:** conceptualization, data curation, formal analysis, investigation, methodology, project administration, resources, validation, writing – original draft, writing – review and editing. **Mahtab Motevasselian:** conceptualization, data curation, formal analysis, methodology, project administration, resources, supervision, writing – review and editing. **Mohsen Khaleghian:** conceptualization, data curation, methodology, writing – review and editing. **Roya Biglarifard:** data curation, methodology, writing – original draft, writing – review and editing. **Seyed Hamzeh Mousavie:** data curation, formal analysis, writing – review and editing.

## Funding

None of the authors received any funding for this submission.

## Ethics Statement

All content of this research adheres to the ethical guidelines developed by the Committee on Publication Ethics (COPE) during the 2nd World Conference on Research Integrity in Singapore in 2010. All parts of this study meet the Code of Conduct (the Ethics Code) and adhere to the legal requirements of the study country, Iran.

## Consent

Written informed consent was obtained from the patient for publication of this case report and accompanying images.

## Conflicts of Interest

The authors declare no conflicts of interest.

## Data Availability

Data is available via contacting the corresponding author.
